# Triboelectric Contact Localization Electronics: A Systematic Review

**DOI:** 10.3390/s24020449

**Published:** 2024-01-11

**Authors:** Wei Xu, Qingying Ren, Jinze Li, Jie Xu, Gang Bai, Chen Zhu, Wei Li

**Affiliations:** 1 College of Electronic and Optical Engineering & College of Flexible Electronics (Future Technology), Nanjing University of Posts and Telecommunications, Nanjing 210023, China; wxu@njupt.edu.cn (W.X.); rqy@njupt.edu.cn (Q.R.); 2 College of Integrated Circuit Science and Engineering, Nanjing University of Posts and Telecommunications, Nanjing 210023, China; lijinze@njupt.edu.cn (J.L.); jiexu@njupt.edu.cn (J.X.); baigang@njupt.edu.cn (G.B.); zhuchen@njupt.edu.cn (C.Z.)

**Keywords:** touch positioning, trajectory detection, triboelectric sensor, self-powered devices, human-machine interface

## Abstract

The growing demand from the extended reality and wearable electronics market has led to an increased focus on the development of flexible human-machine interfaces (HMI). These interfaces require efficient user input acquisition modules that can realize touch operation, handwriting input, and motion sensing functions. In this paper, we present a systematic review of triboelectric-based contact localization electronics (TCLE) which play a crucial role in enabling the lightweight and long-endurance designs of flexible HMI. We begin by summarizing the mainstream working principles utilized in the design of TCLE, highlighting their respective strengths and weaknesses. Additionally, we discuss the implementation methods of TCLE in realizing advanced functions such as sliding motion detection, handwriting trajectory detection, and artificial intelligence-based user recognition. Furthermore, we review recent works on the applications of TCLE in HMI devices, which provide valuable insights for guiding the design of application scene-specified TCLE devices. Overall, this review aims to contribute to the advancement and understanding of TCLE, facilitating the development of next-generation HMI for various applications.

## 1. Introduction

The rising consumer demand for enriched and immersive experiences in wearable devices, coupled with a burgeoning need across diverse industries such as gaming, healthcare, and education, has spurred a surge in research devoted to extended reality (XR) technologies [1] and wearable electronics [2,3,4]. The objectives of XR technologies are to deliver multiple sensations, including vision, hearing, touch, temperature, etc., to the user without perception distortion and collect the user’s multi-modal input via the human-machine interface (HMI) [5]. The seamless interaction and immersive experiences of the XR system heavily rely on the low latency and high accuracy recording of the user’s gesture, body movement, eye movement, voice, and facial expression [6,7]. The current challenges lie in seamlessly integrating with non-flat surfaces, conforming to the contours of the human body, and achieving a compact and space-efficient design. These challenges necessitate the development of flexible HMI [8]. One of the primary objectives of the intensively studied flexible HMI devices such as touch panels [9], electronic skin (e-skin) [10,11], and the Internet of Things (IoT) [12,13] is to achieve accurate spatial tracking to implement functions like contact detection [14], body movement detection [15,16], and handwriting character recognition [17]. Therefore, contact localization electronics that are crucial in obtaining spatial information have undergone rapid development.

Following the pioneering work of Wang et al. in 2012 [18], who introduced the triboelectric nanogenerator (TENG), the progressive advancements in TENG have demonstrated tremendous potential for application in high-entropy energy collection [19,20]. In addition to energy harvesting, triboelectric sensors also have demonstrated advantages such as low cost, easy fabrication, and a wide range of material options [21,22,23]. These advantages benefit the research on triboelectric contact localization electronics (TCLE). Wang et al. pioneered the construction of TCLEs by developing a 6 × 6 triboelectric sensor matrix for pressure mapping [24] and a 4 × 4 triboelectric sensor matrix for finger touch positioning [25] in 2013. Building on this foundation, subsequent research has introduced TCLEs utilizing rubber [26], plastic [27], and fiber [28] tribo-surfaces, thereby extending their application to various fields such as touch panels, e-skin, smart fabrics, etc. [29,30,31,32]. Furthermore, TCLE utilizes contact-induced triboelectric output for contact localization. Therefore, the triboelectric approach has the potential to reduce energy consumption and battery volume in HMI devices, allowing for the development of lightweight and long-endurance designs [33,34].

**Figure 1:**
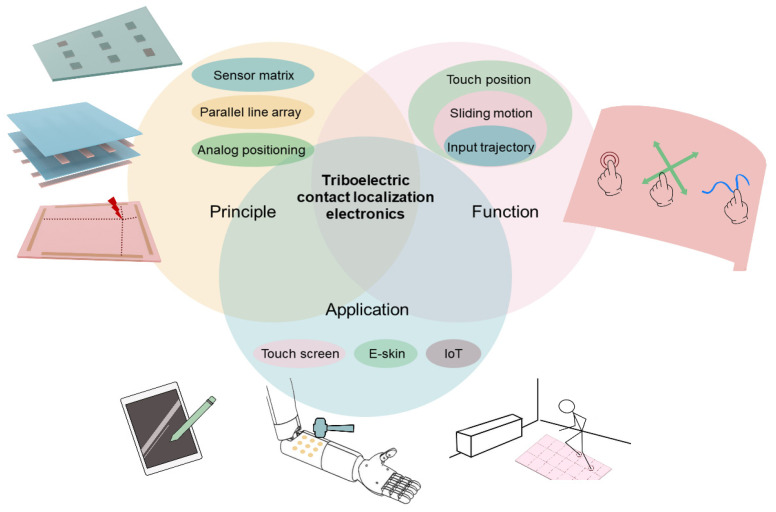
Diagram of representative design principles, functions, and applications of TCLE.

The key challenge of TCLE lies in improving the resolution while simultaneously addressing the issues of decreased accuracy and an increased number of signal channels. To overcome this challenge, novel working principles such as sensor matrix [35], parallel line array [36], and analog localization [37], and signal processing methods such as deep-learning signal processing [38] have been proposed. Additionally, as shown in Figure 1, TCLE has expanded its functionality beyond contact positioning to include sliding detection, input trajectory detection, and handwritten character recognition, stimulating its applications in diverse HMI devices [39,40,41]. The current reviews on triboelectric tactile sensors provide a thorough discussion of the theory, structure, and material aspects of the tactile sensor unit [42,43,44]. However, these reviews predominantly consider TCLE as an application example of the tactile sensor unit array, lacking in-depth analysis of the contact localization principle and design considerations. Therefore, here, we present a systematic review of TCLE with an arrangement as follows: Section 2 discusses the development path of the working principles of TCLE; Section 3 reviews the design considerations and achievable functions of TCLE; Section 4 presents the various application scenarios that have been developed in HMI devices; and Section 5 provides perspectives on future research of TCLE.

## 2. Working Mechanisms

### 2.1. Triboelectric Contact Localization

**Figure 2:**
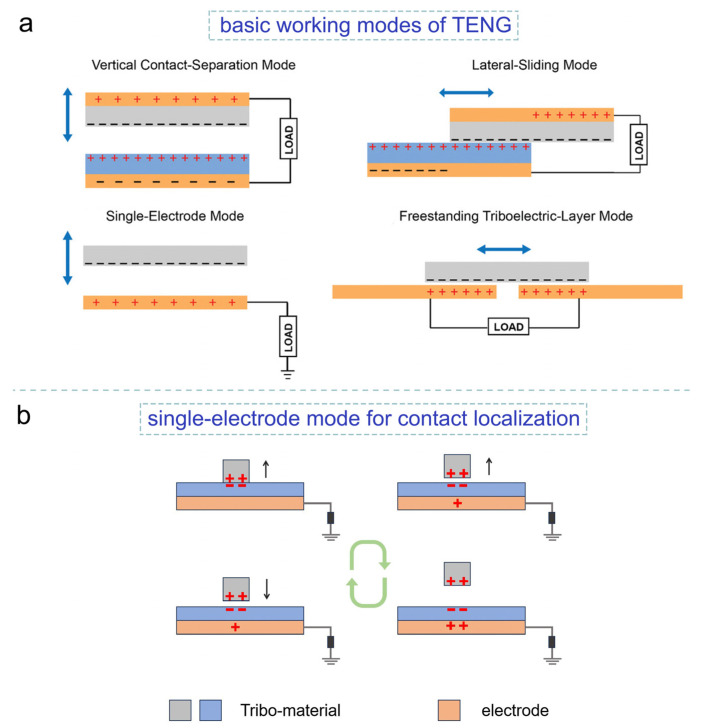
Diagrams of (**a**) the four basic working modes of TENG (reproduced with permission from ref. [46], Copyright 2018, John Wiley and Sons) and (**b**) a working cycle of single-electrode mode TENG for tap detection.

TENG operates on the principle of contact electrification, which involves the transfer of tribo-charges at the contact interface and subsequent electrostatic induction to generate charge flow within the external circuit [45]. The four basic working modes of TENG are shown in Figure 2a [46]. The relative displacement of the opposing tribo-charges on the surfaces of two tribo-materials results in a change in electrical potential at the electrode, subsequently generating current in the connected load.

The typical application scenario of TCLE involves locating the tapping or sliding motions of a moving object, such as a finger. Therefore, the single-electrode mode is highly suitable for TCLE as it eliminates the need to attach an electrode to the moving object. Figure 2b illustrates a typical working cycle of single-electrode mode TENG for tap detection. During a tap motion, a contact-separation process occurs. When two tribo-materials come into contact, the tribo-charges on the TCLE surface become screened, resulting in the transfer of charges from the electrode to the ground. Upon separation, the screening effect diminishes, leading to a reversed current in the connected load. Similarly, during a slide motion, as the moving object overlaps with the electrode position on the TCLE, the tribo-charges on the surface of the moving object screen the tribo-charges on the corresponding surface of the TCLE. This screening effect alters the potential distribution between the electrode and the ground, which can be detected as a signal indicating the slide motion. The V-Q-x governing equation (Equation (1)) proposed by Niu et al. [47]—where *V* is the voltage difference between the electrode and the ground, *C*(*x*) is the equivalent capacitance between the electrode and the ground, *Q* is the transferred charge between the electrode and the ground, and *V_OC_*(*x*) represents the contribution of polarized triboelectric charges to *V*—provides a mathematical understanding of the working mechanism of the single-electrode mode TENG.

(1) $$V=-\frac{1}{C(x)}Q+V_{OC}(x)$$

As illustrated in Figure 2, *V_OC_*(*x*) increases with the separation distance *x* between the tribo-materials, initiating the charge transfer process. This process reconstructs potential balance, generating current pulses upon touch and sliding motion. It is also evident that the location where the tap or slide motion occurs on the TCLE corresponds to the electrode that undergoes a potential change relative to the ground. This fundamental understanding serves as the basis for the design of TCLE.

The high voltage output characteristic of TENG [48] makes TCLE superior to signal strength. By employing appropriate tribo-material engineering, it is possible to achieve peak voltage outputs exceeding 50 V even with subtle contact pressure [49] (0.5 kPa, typical pressure value of finger touch [50]). However, the process of contact-transferred charge accumulation at the contact surface necessitates multiple contacts (typically ranging from a few to dozens) for the tribo-charge density to reach saturation [51,52]. At the unsaturation state, the tribo-signal exhibits a greater variance compared to the saturation state. Additionally, the dissipation of surface tribo-charge [53,54], particularly during long contact time intervals, leads to a change in the surface tribo-charge density and, consequently, affects the output triboelectric signal. Therefore, one of the major challenges for TCLE is to enhance signal consistency, which in turn reduces signal processing complexity and improves detection accuracy. Alternatively, a novel working principle or device design is anticipated to enable precise contact localization, irrespective of the inconsistent triboelectric signal strength.

### 2.2. Working Principles of TCLE

#### 2.2.1. Sensor Matrix

**Figure 3:**
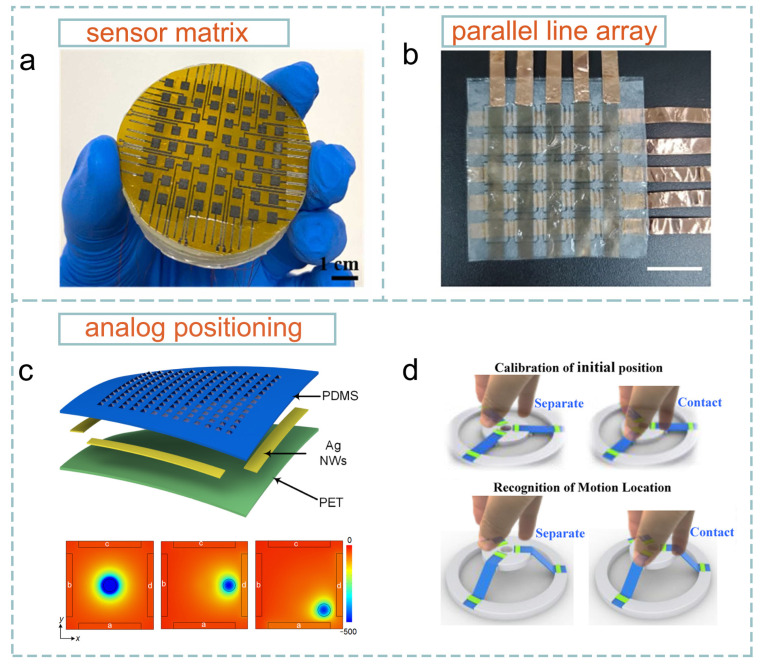
TCLEs utilize different design strategies: (**a**) sensor matrix (reproduced with permission from ref. [55], Copyright 2023, Elsevier); (**b**) parallel line array (reproduced with permission from ref. [58], Copyright 2021, John Wiley and Sons); (**c**) (reproduced with permission from ref. [37], Copyright 2016, American Chemical Society); and (**d**) (reproduced with permission from ref. [59], Copyright 2019, Elsevier) analog localization.

Sensor matrix, parallel line array, and analog positioning design strategies have been successively proposed and have emerged as the primary approaches for TCLE. The sensor matrix design strategy, characterized by its simplicity in working principle, was the initial method employed in TCLE design [24]. Typically, triboelectric sensor units are uniformly distributed across the detection plane. One or more sensor units, decided by the sensor density and the contact area, will be triggered to output a triboelectric signal when contact occurs. By analyzing the coordinates of the triggered sensor units, the precise contact location can be directly determined. As shown in Figure 3a, Guo et al. proposed an 8 × 8 triboelectric sensor matrix for tactile pattern recognition [55]. The sensor unit, consisting of a silicon rubber triboelectric layer and a laser-induced graphene bottom electrode, operates in the single-electrode mode of TENG. Real-time touch positioning can be achieved by mapping the output voltage of the sensor units. The arrangement of sensor units can be tailored to suit diverse application scenes. For instance, Qu et al. [56] strategically positioned three sensor units, employing eutectic gallium–indium liquid metal electrodes and silicone tribo-material, in a triangular configuration on the fingertip. They established a direct correlation between touch positions on the finger and the triboelectric output differences in the three sensor units. This approach successfully achieved precise contact localization among five detection pixels on the fingertip. Moreover, the sensor matrix can be easily assembled using individual sensor units, allowing for a straightforward and convenient device design. Building upon the foundation of a sensor unit characterized by ultrahigh stretchability (1160%) and transparency (96.2% for visible light), which utilized elastomer tribo-material and ionic hydrogel electrodes, Pu et al. [57] introduced an artificial skin with a 3 × 3 sensor matrix. This innovative design enables both single- and multi-contact point localization.

Despite the convenient and flexible device design capability, a notable challenge associated with the sensor matrix strategy is that each sensor unit requires an individual signal line, introducing complexity to the signal processing aspect. Moreover, a substantial number of signal channel poses difficulties in the design of interconnection topology. In Figure 3a, it can be observed that the signal line needs to be routed close to other sensor units, potentially leading to unintended triggering of nearby sensors when the contact area covers the route of the signal line. This issue becomes more pronounced in high localization resolution scenarios.

#### 2.2.2. Parallel Line Array

The parallel line array strategy, similar to the commonly used touch positioning method in commercial touch screens, provides a solution to the aforementioned challenges. Two mutually perpendicular arrays of parallel electrodes are utilized, forming a 2D coordinates system at the cross points of the arrays. When sliding or tapping occurs at these cross points, triboelectric signals are generated in the corresponding electrodes of the two arrays. The wiring for the parallel line array strategy can be conveniently routed on the ends of the electrode, eliminating the need for wiring within the detection area. Moreover, the geometric design of the electrode offers flexibility, allowing for various configurations such as using parallel serpentine lines to enhance stretchability [60]. However, the fundamental principle of contact localization remains unchanged. The work of Pratap et al. [61] exemplifies the effective utilization of the parallel line array strategy. A self-powered keypad was proposed that utilizes a 3 + 3 strip electrode array to locate finger touch within 9 pixels. Nanostructured polydimethylsiloxane was employed to enhance the tribo-output from human skin contact, resulting in a peak voltage signal of ~25 V. As shown in Figure 3b, Lin et al. reported an interface-independent contact localization panel with a 5 + 5 parallel electrode array [58]. A novel structure of two face-to-face TENGs at the internal interlayer was proposed to overcome the material limitation of the top protective layer. Triboelectric signals in the top and bottom electrode arrays determine the contact point within the 5 × 5 detection pixel array.

The double electrode layer structure design of the parallel line array strategy inevitably increases difficulties in device preparation. Nevertheless, to construct a TCLE with M × N detection pixels using the parallel line array strategy, only M + N signal channels are required. This highlights the advantages of the parallel line array strategy, such as a significantly reduced signal channel complexity and simplified wiring, underscoring its substantial potential for use in TCLE.

#### 2.2.3. Analog Localization

Researchers continue to make progress in exploring novel design strategies, further simplifying device design and improving contact localization resolution. The analog positioning strategy was first proposed by Shi et al. for a self-powered smart skin [37] (Figure 3c). A voltage ratio, specifically the electrostatic induction voltage ratios of opposite electrodes positioned on the four sides of the detecting plane, was established to correspond to contact positions. This allows the determination of the contact position by measuring the voltage ratios on two pairs of electrodes and referencing a ratio-position look-up table. Consequently, only four signal channels are required to achieve touch positioning with an average resolution of 1.9 mm. Building on this approach, Zhang et al. proposed a GPS-inspired stretchable electronic skin to further reduce the number of signal channels to 3 [62]. Touch positioning with an average error sum of ~ 1 mm was achieved. To explore the function-driven designs utilizing the analog positioning strategy, Chen et al. proposed a nano-manipulation terminal for controlling the objects in three-dimensional space [59]. As shown in Figure 3d, the length changes of the strip can be determined by measuring the signal ratios from the two terminal electrodes. This enables the localization of the mobile object that connects three strips.

In the quest for superior contact localization resolution, the analog positioning strategy stands out with the highest theoretical resolution among the three discussed strategies. As outlined in Table 1, the challenges of wiring complexity and signal channel augmentation become more pronounced with the expansion of sensor units in both the sensor matrix and parallel line array strategies, essential for achieving higher resolution. Consequently, there exists a constraint on sensor density or line electrode density that imposes limitations on attainable resolution. Nevertheless, it is important to note that the analog positioning strategy is not exempt from challenges, as the environmental noise can introduce measurement error in the form of deviation between the measured tribo-voltage ratio and the reference ratio in the ratio-position look-up table [63,64]. Moreover, the validity of the pre-determined ratio-position look-up table is restricted to the present device configuration, indicating that recalibration of the look-up table is required in the event of device deformation. Timely recalibration poses challenges for wearable HMI where device deformation occurs frequently. Therefore, continuing research work is anticipated to improve the robustness of the analog positioning strategy. 

## 3. Potential Functions Enabled by TCLE

### 3.1. Touch Positioning

**Figure 4:**
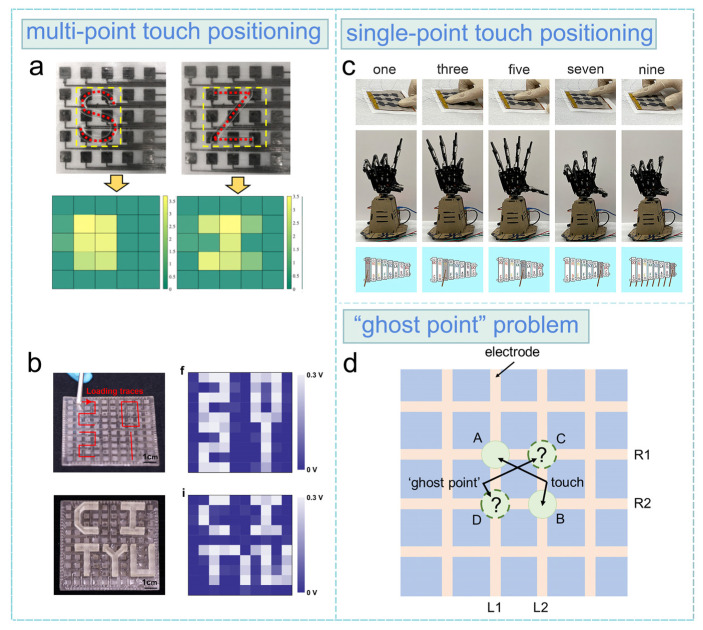
(**a**) All-printed sensor matrix for triboelectric multi-point contact localization (reproduced with permission from ref. [67], Copyright 2020, Elsevier). (**b**) 3D-printed TENG sensor matrix (reproduced with permission from ref. [68], Copyright 2023, KeAi Communications Co. Ltd.). (**c**) Rough surface-enhanced triboelectric tactile sensor array using parallel line array design strategy (reproduced with permission from ref. [69], Copyright 2023, American Chemical Society). (**d**) Schematic sketch of the ‘ghost point’ problem.

Based on the device design strategies discussed in Section 2.2, advanced functions, including touch positioning, slide detection, and slide trajectory detection have been successfully achieved. Single-point and multi-point touch positioning is crucial for the touch operation of the keypad, touchpad, and e-skin [65,66]. The sensor matrix strategy is commonly employed to achieve the multi-point touch positioning function, owing to the independent signal lines of each sensor unit. As shown in Figure 4a, a 5 × 5 matrix consisting of all-printed triboelectric sensors and wrinkled silver electrodes was proposed by Li et al. for the detection of the multi-point contact of a plastic plate [67]. To address the issue of crosstalk between sensor units, Li et al. [68] proposed a 3D-printed TENG sensor matrix (size: 7.5 cm × 7.5 cm, Figure 4b) with 100 sensor units. The 3D patterned substrate effectively enhances independence between sensor units and suppresses the maximum crosstalk output to 10.8%.

Parallel line array strategy can be utilized for single-point contact detection. Figure 4c depicts the 3 × 3 cross-interaction tactile sensor array proposed by Zhang et al., which utilizes a MXene electrode and rough surface to enhance electrical output for single-point touch positioning [69].

However, when applying the parallel line array strategy, attention should be paid to the ‘ghost point’ problem that is also encountered in self-capacitance type capacitive touchpads [70]. In Section 2.1, as discussed earlier, contact at the electrode area produces a triboelectric voltage/current output. In the parallel line array design, detectable points are situated at the cross points of electrodes in two layers. The contact-induced output can be identified at both ends of the electrode. During operation, the output signals are scanned in the two electrode layers, and an electrode with output in both layers determines one of the contact position coordinates in a 2D system. However, the presence of “ghost points” can compromise accurate positioning. Figure 4d illustrates this issue. In a scenario where simultaneous touches occur at points A and B, triboelectric signals are generated in electrodes R1, R2, L1, and L2. Consequently, the obtained 2D coordinates are (R1, L1), (R1, L2), (R2, L1), and (R2, L2). The emergence of “ghost points” (R2, L1) and (R1, L2) results in incorrect localization. While fully synchronized touch is improbable in practice, there exists a small time interval between contacts at two points. This temporal distinction leads to two discernible triboelectric pulse peaks in the output signals, offering a potential solution to this issue through judicious signal processing.

### 3.2. Slide Detection

During the sliding motion, the TCLE and the moving object are constantly in contact. As discussed in Section 2.1, even while maintaining contact, the sliding of the moving object across the electrode leads to the generation of a triboelectric signal. Consequently, by employing a rationally designed electrode pattern, the trajectory points during the slide can be recorded to retrieve the spatiotemporal location or restore the sliding trajectory. This understanding paves the way for the realization of diverse advanced functions, such as slide direction detection, slide localization, and handwriting trajectory recognition [66,71,72].

**Figure 5:**
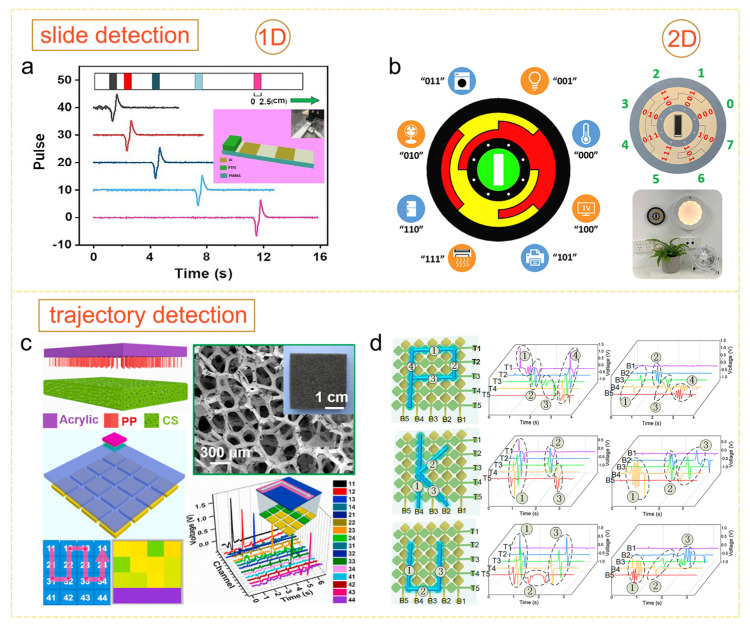
(**a**) Triboelectric sliding detection bar with the 1D electrode array (reproduced with permission from ref. [73], Copyright 2020, John Wiley and Sons). (**b**) Gary code-based sliding direction detection panel (reproduced with permission from ref. [39], Copyright 2020, Elsevier). (**c**) Sliding trajectory detection panel based on sensor matrix design strategy (reproduced with permission from ref. [74], Copyright 2020, Elsevier). (**d**) Self-powered e-skin for sliding trajectory detection based on parallel line array design principle (reproduced with permission from ref. [75], Copyright 2020, American Chemical Society).

The spatiotemporal sliding trajectory dot sequence can be recorded as a sequence of triboelectric pulses. By utilizing a customized electrode topology, both 1D and 2D slide detection can be achieved. As shown in Figure 5a, Yang et al. proposed a sliding detection TENG utilizing the polytetrafluoroethylene tribo-material and one-dimensional distributed Al electrodes [73]. By extracting the timing information of the triboelectric pulse on each electrode, 1D dynamic addressing of the stylus is achieved. Based on the results obtained from the 1D electrode array design, it can be inferred that the width and interval of the triboelectric pulses align with the width and interval of the electrodes. Hence, by employing a combination of 1D electrode arrays spanning various directions, it becomes possible to detect sliding directions through the characteristics of the triboelectric pulse sequence. As shown in Figure 5b, Qiu et al. proposed a control interface based on a gray code-inspired Cu electrode pattern [39]. Each sliding direction is associated with a distinctive combination of electrode array widths. Consequently, by considering the time node combination of the triboelectric pulses generated when the finger reaches each electrode, it becomes feasible to determine the sliding direction. Guo et al. adopted a similar design concept and proposed a soft HMI with an Archimede spiral electrode configuration. This design covers all possible sliding directions and successfully achieves the detection of multi-directional sliding movements [40].

By continuously recording the tracing point with TCLE, the handwriting trajectory can be restored for handwriting digits and character recognition. The sensor matrix design strategy is the optimal approach to realize tracing point detection with high accuracy and high reliability. Figure 5c shows the trajectory detection panel proposed by Cui et al. [74] which utilizes the conductive sponge electrode unit. The act of sliding the polypropylene brush on the sponge electrode units produces triboelectric signals, allowing for the restoration of the trajectory by addressing the activated sensor units. When applying the parallel line array strategy, the intersection points of the two line arrays correspond to the detectable sites. Simultaneous sliding-generated triboelectric pulses in the two electrode arrays indicate the presence of sliding at the detection site. However, the recognition accuracy may experience a decrease for certain trajectories. This decrease may occur when using a rectangular electrode, as the triboelectric signal strength may be too weak to be identified when sliding along the electrode. This is due to the limited variation in the overlap area between the moving object and the rectangular electrode. Therefore, pattern engineering is required to ensure the output strength. As shown in Figure 5d, Guo et al. proposed a self-powered e-skin utilizing the polydimethylsiloxane tribo-material and silver nanowire electrode [75]. The “line” electrode incorporates five quadrilateral valves to ensure that when the finger slides along it, there is a sufficient variation of the overlap area between the finger and electrode. By scanning the triboelectric signals on each electrode, the sliding trajectory is successfully extracted based on the output map. In addition to pattern engineering, working mode optimization is also a feasible approach for robust device designs. The self-powered trajectory-tracking panel proposed by Ba et al. [41] utilizes a sensor unit that combines two TENGs working in the contact-separation mode and single-electrode mode, respectively. The independent triboelectric signal excitation in two TENGs ensures signal strength in both the top and bottom signal output channels. It is also worth mentioning that the crosstalk reduction effect owing to the electrode miniaturization design discussed in Ref. [41] is valuable for the reference of TCLE, especially in cases with high sensor unit density.

## 4. Applications of TCLE

Touch positioning, sliding motion detection, and sliding trajectory detection functions of TCLE are indispensable for enabling the touch operation of HMI [76,77], tactile sensation of E-skin [78,79], and human behavior detection of IoT [80,81]. Additionally, the energy-saving ability of TCLE is anticipated to substantially enhance the lightweight and long-endurance design of wearable/portable HMIs, such as foldable screens, smart home devices, and flexible keystrokes. Therefore, the extensive research conducted on TCLE has resulted in the discovery of various applications in this field, such as HMI for robot control [82,83,84]. Moreover, there has been a surge in interdisciplinary research on artificial intelligence (AI) and tribo-electronics [85]. The signals obtained from TCLE not only encompass position information but also contain valuable data that reflects user habits and other relevant features. Numerous studies have presented evidence that AI can effectively extract these input features to realize advanced functions such as user identification [7,86].

### 4.1. HMI

**Figure 6:**
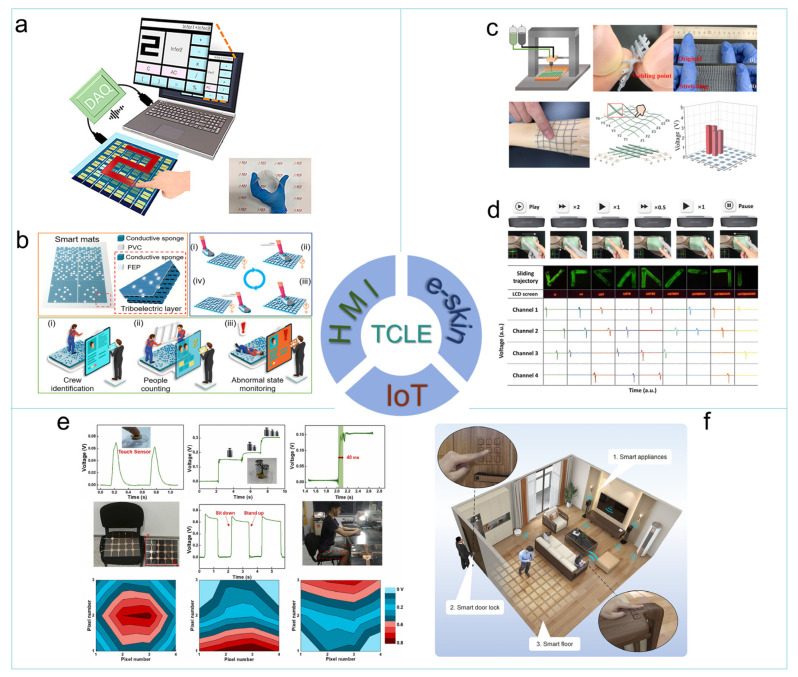
Applications scenes of TCLE: (**a**) flexible touchpad for handwriting digit recognition (reproduced with permission from ref. [89], Copyright 2020, Elsevier), (**b**) self-powered smart mat system for crew identification (reproduced with permission from ref. [90], Copyright 2022, American Chemical Society), (**c**) stretchable smart mesh for contact localization (reproduced with permission from ref. [91], Copyright 2021, Elsevier), (**d**) touch operation panel with sliding trajectory detection function (reproduced with permission from ref. [92], Copyright 2022, the American Association for the Advancement of Science), (**e**) smart mat for sitting posture recognition (reproduced with permission from ref. [93], Copyright 2022, Elsevier), and (**f**) wood-based self-powered IoT devices for smart home application (reproduced with permission from ref. [94], Copyright 2022, American Chemical Society).

Owing to the high accuracy and robust performance of AI-based image and signal recognition [87,88], AI techniques are widely used for the realization of handwriting character recognition and user identification of TCLE. As shown in Figure 6a, Yun et al. proposed a transparent flexible touch panel with a handwriting digit recognition accuracy of 93.6% and 91.8% at the bending angles of 0° and 165°, respectively [89]. The recorded input trajectory was classified by a pre-trained neural network utilizing the training samples from the Modified National Institute of Standards and Technology database (MNIST). Figure 6b shows the self-powered smart mats proposed by Wang et al. [90] utilizing conductive sponges with different filling rates. This approach allows for differentiated triboelectric signal strength and contact position to be determined from the step-detection units of a 3 × 2 sensor array. A CNN was then used to realize crew identification by learning and identifying the walking gait patterns, which is a video privacy-protected approach for security surveillance. These achievements indicate that the application of AI techniques in TCLE promises enhanced convenience and effectiveness owing to the developing AI platform and computing power on portable devices.

### 4.2. E-Skin

E-skin serves as a crucial platform for realizing the tactile perception of artificial limbs [95], intelligent robots [96], and wearable HMI terminals [97]. Achieving conformality and ensuring optimal wearing comfort are critical considerations in the application of TCLE in e-skin. Addressing these concerns requires further research in advanced materials and the development of novel fabrication methods. Based on a 3D-printed fiber composition comprising a sheath made of PTFE + PDMS mixture and a core consisting of PDMS + graphene mixture, a smart textile grid with good stretchability, breathability, and washability was proposed by Chen et al. [91] As shown in Figure 6c, a 6 × 6 contact localization mesh that can be easily attached to the skin and curved object surface was constructed utilizing the parallel line array strategy. To explore the system integration method, Zhao et al. proposed a touch operation panel [92] based on the sensor matrix strategy (Figure 6d). Sliding trajectory recognition is achieved by a 2 × 2 sensor matrix and a programmable control system consisting of data acquisition, signal processing, and operation control modules, which sets a high standard for system-level TCLE application.

### 4.3. IoT

The wide range of material options, combined with the cost-effective advantages of TENG [98], offers promising application prospects for TCLE in the low power consumption design of wireless IoT devices [99]. As shown in Figure 6e, Lei et al. proposed a sitting posture monitoring mat with a self-powered sensor incorporating a bamboo-inspired dielectric layer for remote healthcare [93]. The unique pressure distribution associated with the sitting posture can be identified by analyzing the voltage output map generated by the sensor array. Due to the larger size of the device, the large-scale and cost-effective fabrication methods pose a crucial challenge in the design of IoT for smart home applications. Shi et al. reported a simple treatment strategy for wood to prepare the triboelectric material, allowing for the TENG sensor units to be easily integrated with household facilities [94]. As shown in Figure 6f, smart floor and smart door locks were proposed utilizing the senor matrix design principle. By continuously recording the contact position of the human body and the smart floor, it becomes possible to detect the walking route as well as identify instances of falling events.

Table 2 provides a summary of the characteristics of three key aspects (size, stretchability, and resolution) of TCLEs across various application scenarios. First, in the application of IoT, such as smart floors, furniture, and health monitoring equipment, a larger device size is frequently required. A device size exceeding 1 m^2^ has been reported in ref. [100], which proposes a smart carpet for footstep detection. Due to the considerable device size, cable installation poses a manageable challenge in this scenario. However, ongoing efforts to enhance the preparation process are expected to streamline the production of larger devices. 

Next, for wearable devices, flexibility and stretchability of TCLE are required to ensure that the device does not affect the body’s movement. The stretchability of the sensor unit of 200% in ref. [101], which surpasses the stretch limit of human skin, meets the requirement in the design of e-skin. It is also necessary to ensure the breathability, wearing comfort, and lightweight design of the device. The implementation of the ultra-thin, ultra-soft design is a potential path towards achieving a “wear without feeling” TCLE for e-skin. Moreover, the application of e-skin often demands wireless real-time signal transmission to eliminate the need for a rigid signal processing unit and to ensure the comfort of the e-skin.

Lastly, spatial resolution is an important performance indicator of TCLE, especially for touch panel applications. The average area occupied by a single sensor unit in a sensor matrix or a detection pixel in a parallel line array is utilized to represent the resolution. A resolution of 1.25 cm × 1.25 cm is achieved through a sensor matrix design, while a resolution of 1.6 cm × 1.6 cm is achieved through a parallel line array design. There exists a notable disparity when compared to the millimeter-range resolution of commercial capacitive touch screens [103]. A high-precision electrode preparation method is anticipated for the design of high-resolution TCLE. Meanwhile, with the substantially increased resolution, challenges in cabling are anticipated to escalate. Consequently, additional research is warranted to explore signal channel reduction and signal processing methods tailored for high signal line density conditions.

## 5. Overview and Future Perspectives

The practical design strategies of TCLE including sensor matrix, parallel line array, and analog positioning have been established over the last decade. The sensor matrix strategy demonstrates advantages in convenient and flexible device design capabilities. In contrast, the parallel line array strategy presents benefits such as significantly reduced signal channel complexity and simplified wiring. Meanwhile, the analog positioning strategy showcases advantages associated with the highest theoretical resolution. These design strategies pave the way for the development of devices that can perform various functions such as touch positioning, slide detection, and trajectory detection. TCLEs have gained research attention in the field of HMI due to their cost-effectiveness, ease of fabrication, and the availability of a wide range of material options. In addition, their energy-saving characteristics make them suitable for use in power consumption-restricted wearable electronics. Furthermore, the combination of TCLEs with AI techniques has the potential to enhance detection accuracy while offering advanced functionalities, such as user identification.

To ensure the attainment of optimal and robust performance, as well as the development of practical devices, extensive research endeavors are anticipated in the following domains:

(1) In the development of the contact localization principle, the parallel line array strategy shows great potential in practical applications due to its manageable signal channel and cabling difficulties. Ongoing advancements in signal channel reduction methods are anticipated to further simplify signal processing and reduce power consumption. Conversely, the analog positioning strategy finds utility in specific scenarios where ultra-high resolution is essential; however, it necessitates noise-resistant and environment-insensitive designs to ensure robust performance under adverse working conditions.
(2) In the function development of TCLE, a deeper exploration of the combination of TCLE and AI techniques might further improve the robustness and functional diversity of TCLE.
(3) In TCLE applications, a flexible selection of the working principle is recommended, grounded in a deep understanding of the characteristics of different TCLE working principles and specific application requirements. Material engineering should align with application-specific needs, such as wearability, lightweight, and transparency, while innovative device designs are crucial for addressing challenges like cabling difficulties. Ongoing improvements in detection resolution, with an expected attainment of the millimeter level, are poised to open avenues for TCLE in commercial electronic applications. Simultaneously, large-area fabrication methods, coupled with corresponding device design optimizations, also offer significant practical value.

## Figures and Tables

**Table 1:** Overview of the working principles of TCLE.

Working Principle	Signal Channels	Wiring	Main Error Source
Sensor Matrix	Large	Complex	Mal-triggering of sensor units
Parallel Line Array	Moderate	Simple	-
Analog Localization	Small	Simple	Noise, device deformation

**Table 2:** Summary of characteristics of TCLEs for different applications.

Application Scene	Size	Stretchability	Resolution	Ref.
Touchpad	10 cm × 10 cm	-	1.25 cm × 1.25 cm	[35]
Touchpad	8 cm × 8 cm	-	1.6 cm × 1.6 cm	[75]
e-skin	1 cm × 1 cm (a unit cell)	200%	-	[101]
e-skin	6 cm × 6 cm	130% (for a sensor unit)	1.4 cm × 1.4 cm	[102]
Footstep detection mat	1.0 m × 1.15 m	-	11 cm × 11cm	[100]
Smart floor	40 cm × 60 cm	-	8 cm × 12 cm	[13]
